# Sport-Specific Outcome Measures Improve Clinical Assessment of Shoulder Injury in Swimmers: A Cohort Study of Specific PROMs

**DOI:** 10.1177/19417381261431350

**Published:** 2026-03-31

**Authors:** Diogo Nunes Sousa, Tiago Velho, Rui Escaleira, Daniel Moedas, Bárbara Campos, João Felipe Medeiros-Filho, Hélder Pereira, Eduardo Malavolta, Nuno Sevivas

**Affiliations:** † Life and Health Sciences Research Institute (ICVS), School of Medicine, University of Minho, Braga, Portugal; ‡ ICVS/3Bs - PT Government Associate Laboratory, Braga/Guimarães, Portugal; § Surgery Department, ULSAM Alto Ave, Viana do Castelo, Portugal; ‖ Portuguese Swimming Federation; ¶ Shoulder and Elbow Unit, Orthopaedics Department, Hospital CUF Tejo, Lisboa, Portugal; # Universidade Federal do Rio Grande do Norte Natal, RN, Brazil; ** Orthopaedics Department, ULSAM Póvoa de Varzim/Vila do Conde, Póvoa de Varzim, Portugal; †† Ripoll y De Prado Sports Clinic: Murcia-Madrid FIFA Medical Centre of Excellence, Madrid, Spain; ‡‡ Hospital das Clinicas HCFMUSP, Faculdade de Medicina, Universidade de Sao Paulo, Sao Paulo, SP, Brazil; §§ HCOR, Sao Paulo, SP, Brazil; ‖ ‖ Shoulder and Elbow Unit, Orthopaedics Department, ULSAM Médio Ave, Famalicão, Portugal; ¶¶ Shoulder and Elbow Unit, Orthopaedics Department, Trofa Saúde Hospital Braga Sul, Braga, Portugal

**Keywords:** patient-reported outcome measures (PROMs), shoulder, sports medicine, swimming

## Abstract

**Background:**

Swimming is among the most widely practiced sports globally. Up to 70% of swimmers report shoulder pain that impairs performance. Patient-reported outcome measures (PROMs) are effective, objective tools for assessing such injuries. The aim of this study was to develop and validate swimming-specific adaptations of established shoulder PROMs (ASES-swim and SSV-swim) and compare their psychometric properties and discriminatory ability with generic existing instruments.

**Hypothesis:**

Swimming-specific PROMs may more accurately assess shoulder condition in swimmers and monitor recovery, thereby enabling improved evaluation of health status and, ultimately, athletic performance.

**Study Design:**

Cohort study.

**Methods:**

A total of 167 athletes registered with the Portuguese Swimming Federation (FPN) during the 2023-2024 season were enrolled. Participants were stratified into asymptomatic group (AG = 92) and injured group (IG = 75). Monthly assessments included training characteristics, injury status, and responses to ASES, SSV, SSV-sport, and newly developed ASES-swim and SSV-swim questionnaires over 6 months.

**Results:**

SSV-swim correlated strongly with SSV and SSV-sport (*r* = 0.769, *P* < 0.001 and *r* = 0.857, *P* < 0.001, respectively). ASES-swim scores were highly correlated with ASES scores (*r* = 0.994, *P* < 0.001). Both SSV-swim and ASES-swim demonstrated higher internal consistency than the nonadapted PROMs (Cronbach alpha = 0.849 and 0.870, respectively). SSV, SSV-sport, and SSV-swim showed moderate discriminatory ability (area under the curve [AUC] = 0.760, 0.809, and 0.851, respectively), whereas ASES and ASES-swim showed excellent discriminatory ability for detecting injury (AUC = 0.967 and 0.970, respectively).

**Conclusion:**

ASES-swim and SSV-swim are a valid, reliable, and easy to administer tool for the assessment of shoulder injury and recovery in swimmers. Swimming-specific PROMs provide clinically meaningful improvements in shoulder injury assessment accuracy.

**Clinical Relevance:**

Swimming-specific PROMs provide clinicians with a sensitive and reliable method for assessing shoulder injury in swimmers, enabling more accurate diagnosis and individualized rehabilitation. Further research should investigate sports-specific PROM adaptations to optimize athlete care across disciplines.

Swimming is one of the most common sports worldwide.^22^ However, despite its low impact activity and low incidence of traumatic injuries, swimming presents unique biomechanical challenges that predispose athletes to overload injuries, particularly in the shoulder.

The repetitive overhead motion characteristic of all 4 competitive strokes (freestyle, backstroke, butterfly, and breaststroke) creates substantial demands on the glenohumeral joint and surrounding musculature.^12,19^

These lesions are prevalent and affect athletic performance substantially. The prevalence of shoulder pain among competitive swimmers may reach 70%, whereas the overall injury prevalence is 34%.^23^

This high prevalence reflects the sport’s distinctive kinetic chain demands, including the need for coordinated scapulothoracic and glenohumeral motion while maintaining body position and propulsion in an aquatic environment.^6^

When an athlete experiences pain or injury, accurate assessment is essential.^27^ Such assessment must be conducted easily and be applicable in a multidisciplinary context.^8^ Accordingly, it is important to design and implement individualized plans tailored to the needs of each athlete. In swimming, however, the assessment and classification of shoulder injuries are challenging and complex.^3^ This limitation is due partly to the lack of comprehensive, methodologically robust epidemiological and biomechanical studies.^12^ Moreover, no definitive diagnostic, classification, or evaluation tools exist currently.

Monitoring shoulder injuries in swimmers often relies on clinical symptoms, physical examination, and imaging, which provide objective insights into strength, stability, and function. While these tools are essential, these methods are costly, examiner-dependent, and may fail to capture the athlete’s perceived limitations in sport-specific contexts.^35,39^

Patient-reported outcome measures (PROMs) represent validated, standardized instruments that capture patients’ perspectives on their functional capacity, pain levels, and quality of life.

These tools offer several advantages in athletic populations: they are cost-effective, easily administered, free from examiner bias, and provide quantifiable data for monitoring recovery trajectories.^7,16,28,37,40^ They are therefore simple and practical tools that enable assessment of the patient’s perception of functional capacity. PROMs have also been used widely in various sports to monitor recovery, guide return-to-play decisions, and evaluate treatment outcomes.^9^

Given the specificity of the sports population, the Subjective Shoulder Value for Sport (SSV-sport) was recently developed as a score focused on sports performance and has proven to be more effective, practical, and reliable for evaluating the capacity of these athletes.^7^

Recent studies have applied PROMs to monitor swimmer injuries.^33^ However, these instruments do not account for the unique features of each swimming stroke or other intrinsic and extrinsic factors that influence the severity and impact of injury. This study therefore aimed to evaluate the applicability of the American Shoulder and Elbow Surgeons Shoulder Score (ASES),^28^ the Subjective Shoulder Value (SSV),^10^ and the SSV-sport to the context of this specific sport,^7^ and to compare them with PROMs designed specifically for swimming.

More specifically, our objectives were threefold. First, to apply and validate commonly used PROMs for swimmers, including the ASES, the SSV, and the SSV-Sport. Second, to develop, apply, and validate new swimming-specific PROMs designed to capture the unique demands of the sport—namely, the ASES-swim and SSV-swim. Third, to compare the discriminative capacity of general PROMs (ASES, SSV, and SSV-Sport) with these swimming-specific PROMs (ASES-Swim and SSV-Swim) in differentiating injured from asymptomatic swimmers. We hypothesized that PROMs tailored to swimming would assess shoulder condition in swimmers and monitor their recovery more effectively, thereby supporting better evaluation of health status and, ultimately, performance.

## Methods

### Ethical Approval

The study was approved by the Ethics Committee of the Life and Health Sciences Research Institute (Supplementary File I, available in the online version of this article). All participants provided informed consent. Participants could withdraw from the study at any time.

### Type of Study

A prospective cohort study was conducted over 6 months, involving competitive swimmers registered with the Portuguese Swimming Federation (FPN) during the 2023-2024 season (October 2023 to June 2024).

This study was reported in accordance with the Strengthening the Reporting of Observational Studies in Epidemiology (STROBE) statement.^38^

### Procedure

All athletes completed an initial questionnaire that included demographic, anthropometric, training, competition, and injury history data (Supplementary File II, available online). Swimmers were asked to complete a monthly questionnaire to report their training and competition diaries, as well as any pain or injury. Participants were stratified into asymptomatic (AG) and injured (IG) groups.

Athletes were classified according to their competitive level into: state-level swimmers; national-level swimmers; international-level swimmers; elite-level swimmers; masters swimmers.

### Scores Applied

In the questionnaire, athletes answered a section containing PROMs designed to evaluate pain, functional capacity, sport-specific limitations, and the impact of shoulder injuries on daily activities.

These included the ASES,^28^ which assesses pain and limitations in activities of daily living; the SSV,^10^ which captures overall subjective shoulder function and general functional capacity; and the SSV-sport,^7^ which evaluates sport-specific functional limitations, particularly relevant for overhead athletes. The questionnaires were completed once every month. Swimmers were asked to fulfill the form during the first week of each month throughout the 6-month period. To prevent bias, athletes did not have access to their PROMs scores. Detailed descriptions of the different PROMs and respective formulas can be found in Supplementary File III (available online).

### ASES Tailored for Swimming (ASES-Swim)

Adaptation of the ASES was carried out by a multidisciplinary sports medicine team with expertise in swimming. The following tasks were added to ASES: swim crawl, swim backstroke, swim butterfly, and swim breaststroke.

### SSV for Swimming (SSV-Swim)

The SSV was adapted by our multidisciplinary sports medicine team with expertise in swimming.

It comprised 4 questions directed to the 4 swim strokes: crawl, backstroke, butterfly, and breaststroke.

“Regarding the (stroke), how would you rate your shoulder’s overall capacity, with a fully functional shoulder representing 100% and a nonfunctional shoulder representing 0%?”

### Statistical Analysis

Data were analyzed using IBM SPSS Statistics Version 29. Statistical analysis was conducted following the Checklist for Statistical Assessment of Medical Papers (CHAMP statement).^17^

#### Sample

The sample size (n = 167) exceeded the minimum required for statistical power, as determined by an a priori sample size calculation using G*Power (minimum n = 115). This ensured adequate power (>80%) to detect clinically meaningful differences in PROM scores with a significance level of α = 0.05.

#### Test-Retest Reliability

Test-retest reliability was assessed in athletes who maintained their condition for 2 consecutive months. Intraclass correlation coefficients (ICC) for a single rater, absolute agreement, a 2-way random-effects model, and 95% CIs were calculated to assess consistency. Reliability was classified as “excellent” (>0.90), “good” (0.75 to 0.90), “average” (0.50 to 0.75), or “poor” (<0.50).^15^

#### Internal Consistency

Internal consistency was evaluated for ASES, ASES-swim, and SSV-swim. Cronbach’s alpha was classified as “excellent” (≥0.90), “good” (0.80 to 0.90), “acceptable” (0.70 to 0.80), or “unacceptable” (<0.70).^36^ Internal consistency was not assessed for SSV and SSV-Sport, as these were single-question questionnaires.

#### Floor and Ceiling Effect

Floor and ceiling effects were calculated as the percentage of athletes with SSV, SSV-Sport, and SSV-Swim scores of 0% and 100%, respectively. Floor and ceiling effects with frequency <15% were considered acceptable.^14^

#### Criterion Validity

Criterion validity was assessed for each PROM. Spearman’s rank correlation coefficients were calculated with 2-sided 95% CIs. Spearman’s correlation coefficient (*r*) was classified as “strong” (>0.70), “moderate” (0.40 to 0.69), or “weak” (0.1 to 0.39).^31^

#### Predictive Validity

Univariate binary logistic regression analysis was conducted to assess the independent predictive role of each PROM with respect to injury occurrence.

Multivariate binary logistic regression was performed, using the backward stepwise method, to identify the final model with the greatest predictive capacity.

#### ROC Curves

ROC curves were generated to assess the discriminative capacity of each score regarding injury presence. The areas under the curve (AUC) for each PROM were calculated. The AUC was classified as “Excellent” (>0.90), “Good” (0.80 to 0.90), “Reasonable” (0.70 to 0.80), “Poor” (0.60 to 0.70), “Very Poor” (0.50 to 0.60), or “No Discrimination” (AUC = 0.50).^24^ The Youden index and corresponding cutoff point were also calculated for each PROM.

#### Equity, Diversity, and Inclusion

This study included an equal number of women and men, representing athletes of various ages, demographics, and competitive levels. The research team followed a multidisciplinary and inclusive approach, comprising medical doctors, physiotherapists, academic researchers, and medical students.

## Results

### Participants

The questionnaire was completed by 167 federated swimmers, 40 of whom were at the regional competitive level, 85 at the national level, 5 at the international level, 4 Olympic athletes, and 33 masters swimmers. Among the interviewed swimmers, 27% (n = 46) reported a history of shoulder injury.

The average age of participants was 26 years (range, 14 to 75 years). The dominant stroke of most swimmers in the study (43%, n = 72) was front crawl, followed by breaststroke (20%, n = 33), butterfly (19%, n = 34), and backstroke (16%, n = 28). Among the swimmers interviewed, 27% (n = 46) reported a history of shoulder injury. Only 4% of the athletes with a history of injury (n = 2, corresponding to 1% of the athletes interviewed) had a history of surgical intervention, and 1 of these underwent surgery during the study.

### Participant Injury Throughout the Study

In the first assessment, 92 athletes (55%) were assigned to AG (n = 92). The remaining 75 (45%) were assigned to IG (n = 75). In the second assessment of the 105 athletes reassessed, 62% (n = 65) maintained their condition, 34% (n = 36) remained injury-free, and 28% (n = 29) did not have their injuries resolved.

### Responses to ASES-Swim and SSV-Swim

**Figure 1.**
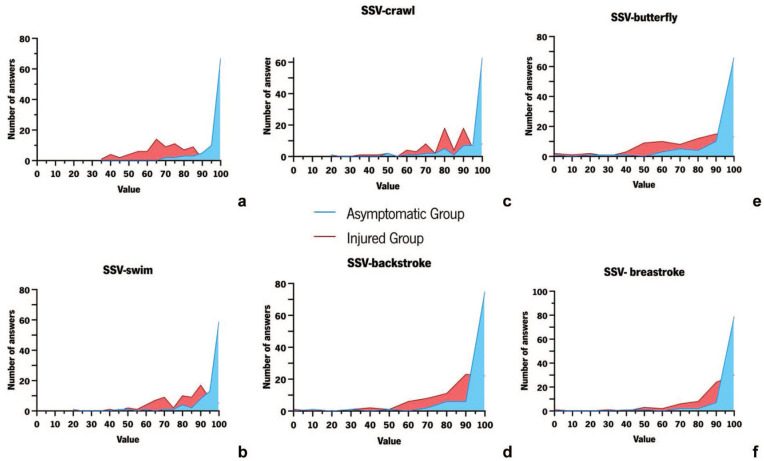
Responses to ASES-Swim and SSV-Swim. (a) Responses to ASES-Swim. (b) Responses to SSV-swim. (c) Responses to SSV-crawl. (d) Responses to SSV-backstroke. (e) Responses to SSV-butterfly. (f) Responses to SSV-breaststroke.

In AG, most participants obtained ASES-Swim scores between 90 and 100 points. In IG, the classification ranged from 35 to 80 points, with a mode of 65 points (Figure 1a).

In AG, the final classification (average) of SSV-Swim presented answers similar to those of the previous PROMs. In IG, the results ranged between 40% and 95% (Figure 1b).

When the same question was applied in a targeted manner to each swimming style, we observed that, in AG, most responses remained between 90% and 100%. However, in IG, the responses differed across swimming styles, reflecting variations in the overall shoulder capacity of these athletes (Figure 1c-f).

### Internal Consistency

#### ASES and ASES-Swim

Evaluation of the internal consistency of the ASES (10 questions) demonstrated an “acceptable” Cronbach’s alpha value (0.786). The ASES-swim evaluation (15 questions) showed a “good” Cronbach’s alpha value (0.870) (Table 1). In both PROMs, the removal of any item did not result in a significant increase in the alpha value.

**Table 1.** Internal consistency

Questionnaire	Number of items	Cronbach’s alpha	Classification
ASES (Section 1 only)	10	0.786	Acceptable
Section 2 (adapted swimming questions)	5	0.763	Acceptable
ASES-Swim	15	0.870	Good
SSV-Swim	4	0.849	Good

ASES, American shoulder and elbow surgeons shoulder score; ASES-Swim, ASES adapted for swimming; SSV, subjective shoulder value; SSV-Swim, SSV adapted for swimming.

Evaluation of the internal consistency of the SSV-swim (4 questions) showed a Cronbach’s alpha value of 0.849 (Table 1). It was also observed that the removal of no items resulted in a significant increase in alpha.

### Reliability of Test-Retest

Test-retest reliability was assessed only in athletes who maintained their condition. All PROMs showed “poor” test-retest reliability in both groups, with ICC <0.50 (SSV: 0.034; SSV-sport: 0.025; SSV-swim: 0.025; ASES: 0.040; ASES-swim: 0.247).

### Floor and Ceiling Effect

The floor and ceiling effects were evaluated for both groups for the different PROMs. No athletes in groups 1 or 2 obtained the minimum classification (0%) in the PROMs. In IG, 20% (n = 15) of the athletes answered the maximum value (100%) on the SSV, indicating an unacceptable ceiling effect. The SSV-sport and SSV-swim exhibited acceptable ceiling effects, with 9% (n = 7) and 4% (n = 3) of athletes responding with the maximum value, respectively.

### Criterion Validity

SSV-swim showed a strong correlation with SSV and SSV-sport (*r* = 0.769, *P* < 0.001 and *r* = 0.857, *P* < 0.001, respectively) (Table 2). The ASES-swim scores correlated strongly with the ASES scores (*r* = 0.769, *P* < 0.001) (Table 2).

**Table 2.** Correlations between the different PROMs with heat map

PROMs	SSV-Sport	SSV-Swim	ASES	ASES-Swim
SSV	0.842* [0.792-0.881]	0.752* [0.677-0.811]	0.414* [0.279-0.532]	0.427* [0.294-0.543]
SSV-Sport		0.825* [0.769-0.868]	0.438* [0.306-0.553]	0.456* [0.326-0.568]
SSV-Swim			0.581* [0.471-0.674]	0.595* [0.488-0.685]
ASES				0.994* [0.991-0.995]
	>0.40 (moderate)	>0.50 (moderate)	>0.70 (strong)	>0.80 (strong)

Data shown as Spearman’s correlation coefficient (*r*) [95% CI]. ASES, American shoulder and elbow surgeons shoulder score; ASES-Swim, ASES adapted for swimming; SSV-Sport, subjective shoulder value for sport; SSV-Swim, SSV adapted for swimming.

* *P* < 0.001.

### Predictive Validity

Univariate binary logistic regression demonstrated that all scores had significant independent predictive capacity, as shown in Table 3.

**Table 3.** Univariate logistic regression of the different PROMs

	*P*	OR	95% CI	*R* ^2^
SSV	<0.001	1.068	1.037-1.100	0.198
SSV-sport	<0.001	1.054	1.031-1.078	0.210
SSV-swim	<0.001	1.090	1.056-1.126	0.298
ASES	<0.001	1.271	1.184-1.365	0.811
ASES-swim	<0.001	1.261	1.177-1.351	0.816

ASES, American shoulder and elbow surgeons shoulder score; ASES-Swim, ASES adapted for swimming; AUC, area under the curve; OR, odds ratio; PROM, patient-reported outcome measure; SSV-Sport, subjective shoulder value for sport; SSV-Swim, SSV adapted for swimming.

In the multivariate analysis with variable selection using the backward stepwise method, ASES-Swim was the only PROM that remained, with odds ratio (OR) = 1.261 [1.177-1.351], *P* < 0.001, and *R*^2^ = 0.816.

### Receiver Operating Characteristic Curves

**Figure 2.**
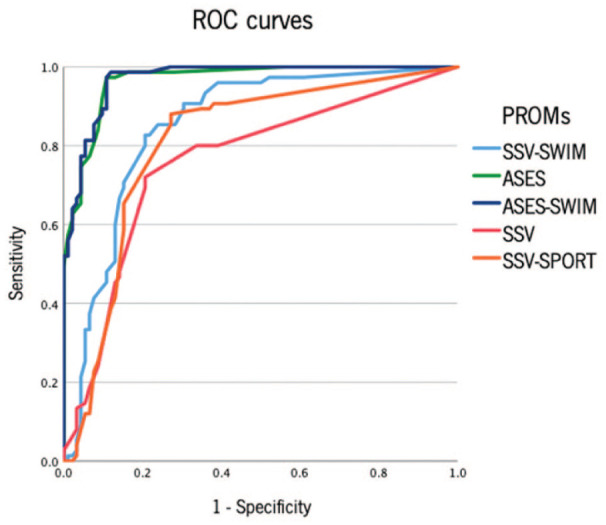
ROC curves from the different PROMs. PROM, PROM, patient-reported outcome measure; ROC, receiver operating characteristic.

Figure 2 presents receiver operating characteristic (ROC) curves from the different PROMs. SSV, SSV-Sport, and SSV-Swim exhibited “moderate” discriminatory ability (AUC values of 0.760, 0.809, and 0.851, respectively), while ASES and ASES-Swim demonstrated “excellent” discriminatory ability (AUC of 0.967 and 0.970, respectively). The lowest cutoff point was for the ASES (85.8), whereas the highest cutoff points were for the SSV and SSV-Sport (94), as shown in Table 4.

**Table 4.** Characteristics of the ROC curve of the different PROMs

	AUC	*P*	CI	Sensitivity, %	Specificity, %	Cutting point
SSV	0.760	<0.001	0.684-0.836	46.7	85.9	94.0
SSV-Sport	0.809	<0.001	0.740-0.879	58.7	84.8	94.0
SSV-Swim	0.851	<0.001	0.791-0.912	56.0	87.0	91.5
ASES	0.967	<0.001	0.943-0.990	92.0	90.2	85.8
ASES-Swim	0.970	<0.001	0.949-0.991	89.3	89.1	88.3

AUC, area under the curve; PROM, PROM, patient-reported outcome measure; ROC, receiver operating characteristic.

## Discussion

The primary conclusion of this study was the confirmation of the hypothesis that PROMs focused on swimming are more reliable and effective in evaluation of shoulder injuries in swimmers. In addition, we demonstrated that both SSV-swim and ASES-swim are consistent, valid, and easily reproducible assessment measures.

### Population

This study involved 167 competitive swimmers, representing a diverse sample of athletes across various ages, specialties, and levels of competitiveness. Including athletes from different competitive contexts is crucial for developing a PROM tailored to their needs.

The inclusion of asymptomatic athletes/patients is unusual in this type of study, since these questionnaires are typically administered before surgical intervention^2,10,11,13,18,34^; however, it is essential to evaluate the populations in the 2 groups to better assess the response trends and predictive capacity of these questionnaires.

### SSV-Swim and ASES-Swim Validation

Our results demonstrate a “strong” correlation between SSV-Swim and SSV, as well as SSV-Sport, and a “moderate” correlation with ASES. ASES-swim exhibited a “strong” correlation with ASES and a “moderate” correlation with SSV and SSV-sport.

The “strong” correlation between the adapted PROMs in the same group (ASES with ASES-Swim and SSV-Swim with SSV) suggests that the adaptation preserved the essence of the questionnaire, validating its use.

The lower, yet still significant, correlation observed between PROMs from different scales reflects the differing response methodologies, which translate to distinct application methods.

### Internal Consistency

The results indicated that the PROMs adapted for swimming, ASES-Swim and SSV-Swim, demonstrated higher internal consistency than their nonadapted counterparts. The SSV-Swim, consisting of 4 items, displayed internal consistency with a Cronbach’s alpha value of 0.849, classified as “Good.” This is particularly noteworthy given the small number of items in the questionnaire.

Both ASES-Swim and SSV-Swim obtained values are comparable with the internal consistency reported for other shoulder-specific PROMs, such as the original ASES and the Simple Shoulder Test.^5,20,26,30^

The increased internal consistency of the adapted PROMs suggests that incorporating modality-specific items enhances reliability, making the questionnaire more suitable for assessing shoulder function in swimmers. The same effect was previously observed in the adaptation of the overuse injury registry “Oslo Sports Trauma Research Center Questionnaire,” where an improvement in the internal consistency of the questionnaire was also observed after its adaptation for sport.^4^

### Floor to Ceiling Effect

The floor to ceiling effect observed in SSV-Swim and SSV-Sport fell between values established as “acceptable”; however, the values observed in the SSV were considered “unacceptable.”^31^

The floor to ceiling effect in SSV-Swim once again highlights how adapting a questionnaire to specific contexts improves the accuracy and reliability of the PROM, as this instrument is better able to capture the limitations and recovery relevant to the sport. This is particularly important in high-functioning populations, like swimmers, where generic PROMs often fail to detect meaningful differences.^29^

### Capacity Test-Retest

The evaluation of the correlation between the responses at the first assessment moment and the second assessment—1 month later—demonstrated an ICC <0.40 in all PROMs applied. These results are distinct from previous work that included SSV and SSV-Sport.^9^

However, it is important to highlight that in the work mentioned above the time between assessments was 2 weeks, while some authors use time intervals as short as 24 hours.^20^

Although there is no consensus on the ideal time between assessments, studies indicate that this should not exceed 3 weeks, since longer time intervals may lead to significant changes in the patient's condition or changes in factors that influence their response (medical support, training load).^41^

The granular nature of the SSV, SSV-Sport, and SSV-Swim scales, which range from 0 to 100%, allows for a broad variation in responses associated with the long interval between evaluations, which may have contributed to the low test-retest consistency observed.

### Predictive Capacity

Univariate logistic regression analysis revealed that all evaluated PROMs exhibited significant independent predictive capacity. However, the PROMs designed specifically for swimmers, SSV-Swim and ASES-Swim, outperformed their nonadapted versions. Notably, ASES-Swim demonstrated the highest *R*^2^ value (0.816), indicating that this questionnaire explains a significantly higher proportion (82%) of cases. The ROC curve analysis further corroborated these findings, with the swimming-specific PROMs—SSV-Swim and ASES-Swim—showing superior discriminatory capacity compared with their nonadapted versions. Both ASES and ASES-swim demonstrated excellent discriminatory capacities (AUC 0.967 and 0.970, respectively). This outcome can be attributed to the multidimensional nature of ASES, which assesses pain, functionality, strength, and the impact on daily activities.

Moreover, the cutoff points differed across PROMs. The lowest cutoff point was observed in ASES (85.8), while the highest cutoff points were identified in SSV and SSV-Sport (94.0). The cutoff points for ASES-Swim and SSV-Swim were 91.5 and 88.3, respectively. These intermediate cutoff points reflect a balance achieved through the adaptation of the questionnaires to the specific needs of sports practice.

### Comparison Between PROMs

The main difference between existing PROMs and those adapted for swimming lies in the activities evaluated. In SSV-Swim, we formulated 4 questions related to different swimming styles, which enabled a more detailed assessment of the impact of injuries on athlete performance. ASES-Swim includes a new section of 5 questions specifically targeting this modality, allowing a better evaluation of the impact of injury on the daily life of these athletes. The data show that SSV-Swim is more capable and accurate in identifying shoulder injuries and monitoring recovery, as both SSV and SSV-Sport tend to overestimate the functional capacity of these athletes.

A further strength is that these swimming-specific questionnaires allow for the discrimination of pain or sensations according to stroke. This stroke-specific differentiation helps doctors and physiotherapists identify movement patterns that exacerbate symptoms, tailor rehabilitation strategies more precisely, and guide return-to-sport decisions based on the demands of each swimming style.

Although our data demonstrate that, as previously described, SSV-sport represents an improvement in relation to SSV,^7^ the use of SSV-Swim allows an even more precise analysis of these injuries, reflecting, once again, the importance of the adaptation being carried out considering specific contexts and daily activities of the population under study.^21^

The difference between ASES and ASES-Swim was not as pronounced as the differences observed between SSV-Swim, SSV, and SSV-Sport. However, our analysis suggests that ASES-Swim represents an improvement over ASES in assessing these athletes. The original version of ASES demonstrated greater predictive ability than any version of SSV. This superiority is attributed to the broader scope of the issues covered, which includes various activities and sections. Finally, while the SSV-Swim and ASES-Swim questionnaires consist of 4 and 15 questions, respectively, it is crucial to evaluate the responses on an individual basis.

As with SSV and SSV-Sport, the SSV-Swim sections, which use free-response scales from 0 to 100, offer more granular responses, which can be advantageous in assessing these athletes, as evidenced in the literature.^1^ Furthermore, the simplicity of applying SSV, SSV-Sport, and SSV-Swim may provide an advantage over ASES and ASES-Swim.

### Clinical Implications

Conventional PROMs fail to assess elite athletes and sports practitioners uniformly and comprehensively, highlighting the need for PROMs adapted to assess postintervention outcomes related to physical conditions and sports demands.^25^ The superior performance of ASES-Swim, even if marginal (ASES-Swim: 0.970; ASES:0.967; difference 0.003), supports the notion that adaptation to the swimming context further optimizes discriminatory performance, particularly in a high-functioning athletic cohort where subtle deficits may be clinically relevant.

Both SSV-Swim and ASES-Swim have proven consistent, concise, and highly capable of assessing shoulder injuries in swimmers. The use of adapted questions reflecting the unique movements of the sport allows for results that more accurately capture the impact of the injury on the activity of the athlete. Although there are no data on the percentage of swimmers requiring surgical intervention compared with those treated conservatively, in our sample of 167 athletes, only 2 (1%) underwent surgical treatment. We suggest that PROMs should be used to assess and monitor interventions in these athletes.

Their ease of interpretation and reproducibility make them ideal for clinical evaluation, contributing to the personalized adaptation of training and recovery plans. The practical advantages of these instruments extend beyond statistical improvements. The concise nature of these questionnaires (15 questions for ASES-Swim and 4 questions for SSV-Swim) supports efficient integration into routine clinical workflows, preparticipation screenings, and return-to-sport assessments.

These validated instruments fill a critical gap in swimming medicine research and practice. Previous studies examining shoulder injuries in swimmers have relied on generic outcome measures or nonvalidated sport-specific questionnaires, limiting the comparability and clinical applicability of findings. The availability of validated swimming-specific PROMs should enhance the quality of future research and enable more meaningful outcome comparisons across studies.

### Future Directions

Despite the key conclusions drawn in this study, further research and clinical applications of these tools are necessary.

Changes in training dynamics, such as updated methodologies or equipment, may necessitate revisiting these scores to ensure they remain current and reflective of injury impact on athlete performance. Advances in technology, such as wearables and tracking applications, can complement PROMs by providing faster, more detailed data for assessing athlete performance and condition.^32^

### Limitations

This study has several limitations. First, it evaluated only 3 previously approved shoulder PROMs among many available scores. Two primary factors guided PROM creation: athletes participation and adherence, both of which were maximized by selecting short, easy-to-answer questionnaires. We used ASES and SSV as bases, which evaluate a broad spectrum of pathologies and interventions. Second, the long interval between assessments may have influenced test-retest results. Third, adherence to the questionnaires decreased throughout athlete monitoring, limiting the number of responses. Finally, while these scores are excellent tools for assessing functional capacity, they do not identify the underlying cause of the injury.

## Conclusion

This study evaluated the capacity and validity of various PROMs in swimming and concluded that swimming-specific PROMs provide clinically meaningful improvements in shoulder injury assessment accuracy. Both SSV-Swim and ASES-Swim have been shown to be consistent, valid, reliable, and easy to administer tools, with a high correlation with previously validated PROMs. Among the PROMs analyzed, ASES-Swim stood out as the most capable, followed by ASES and, subsequently, SSV-Swim.

This reinforces the importance of adapting PROMs to specific contexts. This study also reinforces the value of PROMs in assessing patient conditions—particularly in athletes—emphasizing that tools tailored to specific populations enhance reliability, validity, predictive power, and the accuracy of assessment.

## Supplemental Material

sj-docx-1-sph-10.1177_19417381261431350 – Supplemental material for Sport-Specific Outcome Measures Improve Clinical Assessment of Shoulder Injury in Swimmers: A Cohort Study of Specific PROMs Supplemental material, sj-docx-1-sph-10.1177_19417381261431350 for Sport-Specific Outcome Measures Improve Clinical Assessment of Shoulder Injury in Swimmers: A Cohort Study of Specific PROMs by Diogo Nunes Sousa, Tiago Velho, Rui Escaleira, Daniel Moedas, Bárbara Campos, João Felipe Medeiros-Filho, Hélder Pereira, Eduardo Malavolta and Nuno Sevivas in Sports Health

sj-docx-2-sph-10.1177_19417381261431350 – Supplemental material for Sport-Specific Outcome Measures Improve Clinical Assessment of Shoulder Injury in Swimmers: A Cohort Study of Specific PROMs Supplemental material, sj-docx-2-sph-10.1177_19417381261431350 for Sport-Specific Outcome Measures Improve Clinical Assessment of Shoulder Injury in Swimmers: A Cohort Study of Specific PROMs by Diogo Nunes Sousa, Tiago Velho, Rui Escaleira, Daniel Moedas, Bárbara Campos, João Felipe Medeiros-Filho, Hélder Pereira, Eduardo Malavolta and Nuno Sevivas in Sports Health

sj-docx-3-sph-10.1177_19417381261431350 – Supplemental material for Sport-Specific Outcome Measures Improve Clinical Assessment of Shoulder Injury in Swimmers: A Cohort Study of Specific PROMs Supplemental material, sj-docx-3-sph-10.1177_19417381261431350 for Sport-Specific Outcome Measures Improve Clinical Assessment of Shoulder Injury in Swimmers: A Cohort Study of Specific PROMs by Diogo Nunes Sousa, Tiago Velho, Rui Escaleira, Daniel Moedas, Bárbara Campos, João Felipe Medeiros-Filho, Hélder Pereira, Eduardo Malavolta and Nuno Sevivas in Sports Health
