# Supplementary material for: Sport-Specific Outcome Measures Improve Clinical Assessment of Shoulder Injury in Swimmers: A Cohort Study of Specific PROMs
Source: Sports Health. 2026 Mar 31:19417381261431350. Online ahead of print. doi: 10.1177/19417381261431350 (PMC13038485; doi:10.1177/19417381261431350)
Supplement: sj-docx-1-sph-10.1177_19417381261431350 – Supplemental material for Sport-Specific Outcome Measures Improve Clinical Assessment of Shoulder Injury in Swimmers: A Cohort Study of Specific PROMs [file sj-docx-1-sph-10.1177_19417381261431350.docx]

**Suplementary Table 1.** Characteristics of the participants

The data are presented as frequency and percentage, except for the variable "Age" which is presented as median± interquartile range (mean).

|  | | **Female** | | **Male** | | **Total** | |
| --- | --- | --- | --- | --- | --- | --- | --- |
| Age | | 20±11 (25) | | 20±16 (27) | | 20±14 (26) | |
| Competitive Level | Regional | 25 | 30% | 15 | 18% | 40 | 24% |
|  | National | 40 | 48% | 45 | 54% | 85 | 51% |
|  | International | 1 | 1% | 4 | 5% | 5 | 3% |
|  | Olympic | 3 | 3% | 1 | 1% | 4 | 2% |
|  | Master | 15 | 18% | 18 | 22% | 33 | 20% |
| Dominant Style | Crawl | 33 | 38% | 39 | 47% | 72 | 43% |
|  | Butterfly | 15 | 18% | 18 | 22% | 33 | 20% |
|  | Coasts | 18 | 22% | 10 | 12% | 28 | 17% |
|  | Breaststroke | 18 | 22% | 16 | 19% | 34 | 20% |
| Specialty | Speed | 72 | 86% | 52 | 63% | 124 | 74% |
|  | Mid-Distance | 5 | 6% | 14 | 17% | 19 | 11% |
|  | Bottom | 7 | 8% | 17 | 20% | 24 | 15% |
| Injury history | Yes | 26 | 31% | 20 | 24% | 46 | 27% |
|  | No | 58 | 69% | 63 | 76% | 121 | 73% |
| Types of Injury | Instability | 2 | 8% | 1 | 5% | 3 | 7% |
|  | Dislocation | 2 | 8% | 2 | 10% | 4 | 8% |
|  | Hood Rupture | 3 | 11% | 0 | 0% | 3 | 7% |
|  | Tendinitis | 16 | 62% | 14 | 70% | 30 | 65% |
|  | Other | 3 | 11% | 3 | 15% | 6 | 13% |
| Treatment | Conservative | 25 | 96% | 19 | 95% | 44 | 96% |
|  | Surgery | 1 | 4% | 1 | 5% | 2 | 4% |

**Supplementary Table 2.** Participation throughout the study

The data is presented as "frequency" and "percentage".

|  | | **Female** | | **Male** | | **Total** | |
| --- | --- | --- | --- | --- | --- | --- | --- |
| Month 1 | Total responses | 84 |  | 83 |  | 167 |  |
|  | Group 1 - Asymptomatic | 44 | 52% | 48 | 58% | 92 | 55% |
|  | Group 2 - Injured | 40 | 48% | 35 | 42% | 75 | 45% |
| Month 2 | Total responses | 56 |  | 49 |  | 105 |  |
|  | Group 1 - Asymptomatic | 30 | 54% | 28 | 57% | 58 | 55% |
|  | Group 2 - Injured | 26 | 46% | 21 | 43% | 47 | 45% |
| Month 3 | Total responses | 45 |  | 43 |  | 88 |  |
|  | Group 1 - Asymptomatic | 19 | 42% | 25 | 58% | 44 | 50% |
|  | Group 2 - Injured | 26 | 58% | 18 | 42% | 44 | 50% |
| Month 4 | Total responses | 48 |  | 42 |  | 90 |  |
|  | Group 1 - Asymptomatic | 29 | 60% | 26 | 62% | 55 | 61% |
|  | Group 2 - Injured | 19 | 40% | 16 | 38% | 35 | 39% |
| Month 5 | Total responses | 44 |  | 38 |  | 82 |  |
|  | Group 1 - Asymptomatic | 24 | 55% | 30 | 79% | 54 | 66% |
|  | Group 2 - Injured | 20 | 45% | 8 | 21% | 28 | 34% |
| Month 6 | Total responses | 39 |  | 39 |  | 78 |  |
|  | Group 1 - Asymptomatic | 20 | 51% | 24 | 62% | 44 | 56% |
|  | Group 2 - Injured | 19 | 49% | 15 | 38% | 34 | 44% |
