# Supplementary material for: Sport-Specific Outcome Measures Improve Clinical Assessment of Shoulder Injury in Swimmers: A Cohort Study of Specific PROMs
Source: Sports Health. 2026 Mar 31:19417381261431350. Online ahead of print. doi: 10.1177/19417381261431350 (PMC13038485; doi:10.1177/19417381261431350)
Supplement: sj-docx-2-sph-10.1177_19417381261431350 – Supplemental material for Sport-Specific Outcome Measures Improve Clinical Assessment of Shoulder Injury in Swimmers: A Cohort Study of Specific PROMs [file sj-docx-2-sph-10.1177_19417381261431350.docx]

**QUESTIONAIRE**

# Introduction

The study's general objective is to understand the etiology of shoulder pain in swimmers and identify a predictive model for it.

The data collected in this questionnaire will be used solely for research purposes, respecting confidentiality, and only members of the research team will have access to them. All responses will be kept anonymous and will be destroyed/deleted after the study is completed.

# Study Methodology

The questionnaire is divided into 5 sections:

Section 1: Identification and personal history. (you only have to fill it out once)

Section 2: Competitive specialty. (you only have to fill it out again if there are changes) Section 3: Monthly Training (answer monthly)

Section 4: Shoulder pain (answer monthly)

Section 5: Shoulder Scores (answer monthly)

The study will last 6 months, with 6 questionnaires being administered. The questionnaires should be fulfilled after the last training session of each month.

The questionnaires can be completed until the 9th of that next month. You may withdraw from the study at any time if you so wish.

Your response to this survey is anonymous and confidential and will be analysed together with that of the other respondents. The data will be used for academic purposes only and will be processed in accordance with the General Data Protection Regulation.

# Section 1: Identification

1. Enter your personal code:
2. What’s your height? (centimeters)
3. What’s your weight? (kilograms)
4. Do you have any known medical condition?
5. Have you ever had a shoulder injury?
   1. Yes, surgery
   2. Yes, dislocation
   3. Yes, other

Please specify:_________________

- 1. No

# Section 2: Competition Preferences

1. In what competitive level do you compete currently in?
   1. State Level
   2. National Level
   3. International Level (European championships or World champioships)
   4. Olympic Level
   5. Masters Level (solely competing in Masters Level)
2. What is your preferred stroke?
   1. Butterfly
   2. Backstroke
   3. Breaststroke
   4. Crawl
3. What is the distance you compete the most in?
   1. 50
   2. 100
   3. 200
   4. 400
   5. Distance
   6. Open Water

# Section 3: Training Diary

1. How would you rate your training over the last month?
   1. Normal Load
   2. Taper
   3. Increased Load
2. How many kilometers did you swum per training session?
3. How many meters did you swim with paddles per training session?
4. How many gym sessions did you do per week?
5. How many minutes of warming up out of the water did you do per workout?
6. How many minutes of recovery/stretching did you do per workout?

# Section 4: Shoulder Pain

1. Have you ever had shoulder pain this month?
   1. Yes
   2. Maybe, I'm not sure if it was pain or discomfort
   3. No
2. When do you feel the pain occurs most:
   1. At night
   2. When I wake up, in the morning
   3. During training
   4. After training
3. Do you feel that the pain has affected your performance in training?
   1. It did not affected at all
   2. It affected a little
   3. It affected moderately
   4. It affected a lot
   5. The pain has stopped me from training
4. Do you feel that pain has affected your performance in competition?
   1. It did not affected at all
   2. It affected a little
   3. It affected moderately
   4. It affected a lot
   5. The pain has stopped me from training
5. Which stroke do you feel the greatest intensity of pain in?
   1. Butterfly
   2. Backstroke
   3. Breaststroke
   4. Crawl
   5. None in particular
6. Have you taken any pain medication?
   1. Yes (please specify:_________)
   2. No
7. How would you rate the pain on a scale of 0-10?
8. Did you seek professional help? (physiotherapist/doctor/other)
   1. If you did and you received a diagnosis, please specify:_________.

# Section 5: Shoulder Scores

Section 5.1:

How would you rate your pain on a scale of 0 to 10, where 0 is no pain and 10 is the worst pain imaginable?

A.:_________

Mark the number that best indicates your ability to perform the following tasks:

0 = Unable to perform; 1 = Very difficult to perform; 2 = Something difficult to perform; 3 = No difficulty

1. **Wear a coat**

( ) 0 = Unable to perform;

( ) 1 = Very difficult to perform;

( ) 2 = Something difficult to perform;

( ) 3 = No difficulty.

1. **Sleeping on the affected or sore side**

( ) 0 = Unable to perform;

( ) 1 = Very difficult to perform;

( ) 2 = Something difficult to perform;

( ) 3 = No difficulty.

1. **Washing the back / Tightening the bra**

( ) 0 = Unable to perform;

( ) 1 = Very difficult to perform;

( ) 2 = Something difficult to perform;

( ) 3 = No difficulty.

1. **Perform personal hygiene**

( ) 0 = Unable to perform;

( ) 1 = Very difficult to perform;

( ) 2 = Something difficult to perform;

1. **Comb your hair**

( ) 0 = Unable to perform;

( ) 1 = Very difficult to perform;

( ) 2 = Something difficult to perform;

( ) 3 = No difficulty.

1. **Reach a high shelf**

( ) 0 = Unable to perform;

( ) 1 = Very difficult to perform;

( ) 2 = Something difficult to perform;

( ) 3 = No difficulty.

1. **Lift 4.5 kg above the shoulder**

( ) 0 = Unable to perform;

( ) 1 = Very difficult to perform;

( ) 2 = Something difficult to perform;

( ) 3 = No difficulty.

1. **Throw a ball above the head**

( ) 0 = Unable to perform;

( ) 1 = Very difficult to perform;

( ) 2 = Something difficult to perform;

( ) 3 = No difficulty.

1. **Doing my day-to-day work**

( ) 0 = Unable to perform;

( ) 1 = Very difficult to perform;

( ) 2 = Something difficult to perform;

( ) 3 = No difficulty

1. **Do my everyday sport**

( ) 0 = Unable to perform;

( ) 1 = Very difficult to perform;

( ) 2 = Something difficult to perform;

( ) 3 = No difficulty.

1. **Swim Crawl**

( ) 0 = Unable to perform;

( ) 1 = Very difficult to perform;

( ) 2 = Something difficult to perform;

( ) 3 = No difficulty.

1. **Swim Backstroke**

( ) 0 = Unable to perform;

( ) 1 = Very difficult to perform;

( ) 2 = Something difficult to perform;

( ) 3 = No difficulty.

1. **Swim Butterfly**

( ) 0 = Unable to perform;

( ) 1 = Very difficult to perform;

( ) 2 = Something difficult to perform;

( ) 3 = No difficulty.

1. **Swim Breastroke**

( ) 0 = Unable to perform;

( ) 1 = Very difficult to perform;

( ) 2 = Something difficult to perform;

( ) 3 = No difficulty.

1. **Swim with Paddles**

( ) 0 = Unable to perform;

( ) 1 = Very difficult to perform;

( ) 2 = Something difficult to perform;

( ) 3 = No difficulty.

Section 5.2:

1. How would you rate the overall ability of your shoulder, if a completely normal shoulder represents 100% and a non-functional shoulder represents 0%?

_____________%

2. Regarding sports, how would you rate the overall ability of your shoulder, if a completely normal shoulder represents 100% and a non-functional shoulder represents 0%?

_____________%

3. Regarding the **front crawl**, how would you rate your overall shoulder capacity, if a fully functional shoulder represents 100% and a non-functional shoulder represents 0%?

_____________%

4. Regarding the **backstroke**, how would you rate your overall shoulder capacity, if a fully functional shoulder represents 100% and a non-functional shoulder represents 0%

_____________%

5. Regarding the **butterfly stroke**, how would you rate your overall shoulder capacity, if a fully functional shoulder represents 100% and a non-functional shoulder represents 0%

_____________%

6. Regarding the **breaststroke**, how would you rate your overall shoulder capacity, if a fully functional shoulder represents 100% and a non-functional shoulder represents 0%” The test result was calculated based on the average of the 4 questions.

_____________%
