# Supplementary material for: Sport-Specific Outcome Measures Improve Clinical Assessment of Shoulder Injury in Swimmers: A Cohort Study of Specific PROMs
Source: Sports Health. 2026 Mar 31:19417381261431350. Online ahead of print. doi: 10.1177/19417381261431350 (PMC13038485; doi:10.1177/19417381261431350)
Supplement: sj-docx-3-sph-10.1177_19417381261431350 – Supplemental material for Sport-Specific Outcome Measures Improve Clinical Assessment of Shoulder Injury in Swimmers: A Cohort Study of Specific PROMs [file sj-docx-3-sph-10.1177_19417381261431350.docx]

**Supplementary File II**

**Sport-Specific outcome measures improve clinical assessment of shoulder injury in swimmers: a cohort study of specific PROMs**

## Scores Applied

### In the questionnaire, athletes answered a section containing patient-reported outcome measures (PROMs). These included questions on ASES, SSV, SSV-sport, and swimming-related adaptations of the SSV and ASES. The questionnaires were completed monthly during the first week of each month throughout the six-month period. To prevent bias, athletes did not have access to their PROMs scores.

### American Shoulder and Elbow Surgeons Shoulder Score (ASES)

### The score was calculated based on its original formula:

### ASES = Pain Domain + Daily Life Activity Domain

### Pain Domain = 5 × (10 – Verbal Numeric Pain Scale Score)

### Daily Life Activity Domain = 5 × (Sum of Scores for Questions 1 to 10 / 3)

### Scores range from 0 to 100.

### American Shoulder and Elbow Surgeons Shoulder Score Tailored for Swimming (ASES-swim)

Adaptation was carried out by a multidisciplinary sports medicine team with expertise in swimming. The following tasks were added to ASES: "Mark the number that best reflects your ability to perform the following tasks: 0 = Unable to perform; 1 = Very difficult to perform; 2 = Somewhat difficult to perform; 3 = No difficulty":

1. Swim Crawl
2. Swim Backstroke
3. Swim Butterfly
4. Swim Breastroke

### The score was calculated based on the original formula:

### ASES = Pain Domain + Daily Life/Sports Activity Domain

### Pain Domain = 5 × (10 – Verbal Numeric Pain Scale Score)

### Daily Life/Sports Activity Domain = 5 × (Sum of Scores for Questions 1 to 10 / 4.5)

### Scores range from 0 to 100.

###

### Subjective Shoulder Value (SSV)

### The question asked was: “How would you rate your shoulder's overall ability, with a completely normal shoulder representing 100% and a non-functional shoulder representing 0%?”

### Subjective Shoulder Value for Sport (SSV-sport)

### The question asked was: “Regarding sport, how would you rate your shoulder's overall ability, with a completely normal shoulder representing 100% and a non-functional shoulder representing 0%?”

### Subjective Shoulder Value for Swimming (SSV-swim)

## The SSV was adapted by a multidisciplinary sports medicine team with expertise in swimming.

## It comprised four questions:

## “Regarding the front crawl, how would you rate your shoulder's overall capacity, with a fully functional shoulder representing 100% and a non-functional shoulder representing 0%?”

## “Regarding the backstroke, how would you rate your shoulder's overall capacity, with a fully functional shoulder representing 100% and a non-functional shoulder representing 0%?”

## “Regarding the butterfly stroke, how would you rate your shoulder's overall capacity, with a fully functional shoulder representing 100% and a non-functional shoulder representing 0%?”

## “Regarding the breaststroke, how would you rate your shoulder's overall capacity, with a fully functional shoulder representing 100% and a non-functional shoulder representing 0%?”
